# Therapeutics

**Published:** 1877-08

**Authors:** 


### III. Therapeutics.

A Case of Poisoning by Salicylate of Soda.—F. Petersen. (*Deutsche Med. Wochenschrift*, No. 2 and 3, 1877.)—The case was that of a girl, fifteen years old, who had been operated upon. The operation was a resection of the ankle joint. Fourteen days after the operation, through some misunderstanding, she was given twenty-six grammes of the salicylate in course of twelve hours. The toxic symptoms were similar to those that had been observed in the experiments on animals. The psychical symptoms were very prominent; at times she was perfectly rational, then again, delirious. The ravings were of a melancholy nature. This delirious condition disappeared gradually in the course of eight days, the intervals of sanity increasing. The patient had no remembrance of what occurred in this time. While she was rational she complained of severe headache; could not see well at a distance; suffered from strabismus and excessive mydriasis; had a difficulty in hearing, and a ringing in the ears. The patient was troubled with dysphasia, the author thinks from innervation of n. hypoglossus. The hoarseness lasted four days. Accelerated respiration, forty per minute. The salicylate had no influence on temperature. Of interest are the disturbances of the vaso-motor sytem. In different parts of the body a dilatation of the blood vessels was noticed. Perspiration for a few days was very considerable.

Remedy for Itching.—Bœck. (*L' Union Médicale*, No. 33, 1877.)—The itching which accompanies certain cutaneous affections, such as pruritus, prurigo, and urticaria, constitutes the most painful symptom. To relieve it, the patient is shut up in a box, as for a vapor bath, and under him is placed a box filled with red-hot charcoal, upon which are placed juniper leaves. If they are not fresh they should be moistened with water. The patient is exposed to the vapor every second day for twenty or thirty minutes.

In prurigo, the fumigation succeeds immediately, and several patients treated by this method alone, have left the hospital cured. It has also cured, and without relapses, inveterate cases of pruritus and chronic urticaria. In chronic eczema, and in other cutaneous affections, the effects of fumigation with juniper leaves have not yet been well established. L. W. C.

Antiblennorrhagic Decoction.—Dupouy. (*L' Union Médicale*, No. 46, 1877.)—Four or five grammes (60 to 75 grains) of kava root, a species of pepper from Oceanica, are grated and macerated in a litre of water (one quart nearly), for five minutes, it being shaken several times. It is then filtered, and taken in two doses during the day, either before or after eating, until a cure is effected.

Twenty minutes after the ingestion of the first dose, the patient feels a pressing desire to urinate; but the micturition is already easier and less painful, and the urine has become clear. According to the author, a cure is obtained in ten or twelve days. The kava does not derange the digestive functions, nor produce diarrhœa nor constipation, and stimulates the appetite by its bitter taste. L. W. C.

Mercurial Frictions in the Treatment of Croup.—Foucart. (*Gazette Obstétricale*, 5 May, 1877.)—The author employs mercurial frictions in large doses, and attributes the success which he has had, a greater success than his predecessors have had, who have made use of small doses, to his *modus faciendi*.

In four cases, he has used mercurial frictions in doses of 50 grammes (1.6 ounces) in twenty-four hours. The frictions were made every two hours, alternately around the neck, upon the abdomen, legs, and soles of feet, and the anointed parts were then covered with a layer of wadding. To prevent its action on the gums, he gave at the same time chlorate of potassium. Finally, he prescribed an antimonial potion, which, except in case of imminent suffocation, should only be given 24 or 36 hours after beginning the friction. According to his observations, the mercury had the effect of facilitating the detachment of the false membranes, and of preventing their reproduction by modifying the plasticity of the blood, and this effect was often appreciable in from 24 to 36 hours, as shown by Dr. Simorre, in acute articular rheumatism. An emetic given at the beginning has no action upon the false membranes, which are still too adherent, and it fatigues the stomach, which sometimes becomes refractory to the medicine when its action is the most necessary, that is when the false membranes become loosened.

Of four cases thus treated, two, a boy of three years and a little girl of four years, recovered after vomiting pieces of false membrane and segments of tubes. The other two, girls aged eighteen months and six years, respectively, died, but the author believes they would have recovered if the plan of treatment had been faithfully carried out by the children's parents.

L. W. C.

Salts of Copper in Foods and Drinks.—M. Decaisne. (*L'Union Médicale*, No. 46, 1877.) The author called the attention of the Academie des Sciences, session of 16th April, 1877, to a paper read by him in 1864, on *Absinthe drinkers.* In that paper he said that a large number of the inferior kinds of absinthe possessed all the characters of sulphate of copper, and that a certain proportion of the 150 absinthe drinkers under his notice presented, besides the alcoholism which he was then studying, well-marked symptoms of poisoning by copper.

Fifteen samples of absinthe, collected in the faubourgs of Paris, and analyzed for him, showed, without exception, the presence of sulphate of copper in variable quantities. Some distillers avowed that they added the sulphate of copper for the purpose of coloring it.

Recently, a case reported to him by Dr. Dubest of a young man who had taken a quantity of brandy, presented all the symptoms of acute poisoning by the salts of copper, and life was endangered. This brandy, on analysis, showed the presence of 1.64 grammes of acetate of copper to the litre. The brandy had been distilled in an apparatus which had not been used for a year, and which was charged with acetate of copper.

Dr. Decaisne submits to the Academie the following reflections:

1. That the annals of science, in France and abroad, are full of facts demonstrating poisoning, either acute or chronic, by the salts of copper.

2. That a large number of industries use these salts openly, or for the adulteration of foods and drinks.

3. That improper care of the vessels and utensils employed for industrial and domestic purposes, frequently determines the formation of dangerous salts of copper in greater or less quantities.

4. That the statistics of criminal poisoning in France, from 1857 to 1863, show 110 attempts upon human life by the sulphate and acetate of copper, and assign to these two substances the third rank among poisons used for criminal purposes.

Without wishing to judge of the value of experiments undertaken lately to clear up a question which appears to him improperly stated, he thinks that, in the name of the interests of public hygiene and of justice, it is the duty of hygienists and physicians to counteract the dangerous and fatal tendency to present to the public the salts of copper as almost inoffensive.

L. W. C.

REMEDY FOR WHOOPING-COUGH.—M. Dervieux.—(*Lyon Médical*, No. 11, 1877.) M. Dervieux believes he has found a preservative means in aconite associated with ipecac and cherry laurel water. This mixture is either a veritable preventive, or simply an abortive. His formula is as follows:

| | | | | |
|---|---|---|---|---|
| Extract of aconite, | .05 | grammes | = $\frac{4}{5}$ grain | nearly. |
| Cherry laurel water, | 4. | " | = 1 dram | " |
| Syrup of Ipecac, | 3. | " | = $\frac{3}{4}$ " | " |
| Mucilage, | 200. | " | = $6\frac{1}{2}$ ounces | " |

This is given as soon as the characteristic cough presents itself, in doses of a teaspoonful every hour to young infants; two teaspoonfuls to those more than three years of age, and a teaspoonful to adults every hour. L. W. C.

ERGOT IN ATONY OF THE BLADDER.—*The Doctor*, July 1.—At a meeting of the Berlin Medical Society, Prof. von Langenbeck stated that in atony of the bladder, associated with enlarged prostate, in elderly men, in which the organ is never completely emptied of urine, he has lately tried the hypodermic injection of ergotine with most surprising results. In three cases, the contractile power was at once increased, so as to enable the patient to discharge the additional urine, and in a few days it had so augmented that very little urine was left behind. After one or two injections, the improvement was considerable, and even a diminution in the size of the prostate seemed to have ensued. Dr. Israel said that he had derived the same benefit from the employment of ergotine, and referred to the case of a patient who was thus enabled to hold his water for three hours, whereas before he voided it every ten minutes.

CYANIDE OF MERCURY IN DIPHTHERIA.—Dr. A. Erichsen.—(*Med. Times and Gazette*, April 28.) This remedy is strongly recommended, in minute doses, in diphtheria. Of twenty-five cases treated, only three proved fatal. With its use the membranes become thinner and less adhesive, and even where they had extended into the larynx, threatening obstruction, they had separated, and the larynx again became free. The writer believes that mercury shortens the duration of the diphtheritic process, and that this preparation does not, like the others, disturb digestion or nutrition. To syphilitic children, it may be given for months without disturbance, in doses of $\frac{1}{48}$ of a grain three times daily. The ordinary dose for a child under three years of age, is $\frac{1}{96}$ of a grain, once every hour or two.

The following is the formula:

℞.

| | |
|---|---|
| Hydrargyri cyanatis, . . | gr. j. |
| Aquæ destill. . . . . | ℥vi. |
| Syr. simplicis . . . . | ℥ss. |

M. S. Half or a whole teaspoonful every hour.

Ergot in Urethral Hemorrhage.—Boyland.—(*American Journal of the Medical Sciences*, July, 1877.) "Few anatomical structures are more delicate than the small urethra; few membranes are more highly organized in their physiological action, or more susceptible to pathological process. Reaction, therefore, follows with the utmost facility; indeed, the sensitiveness is so extreme in certain cases requiring antiphlogistic treatment, that the mildest injection is at times productive of urethral hemorrhage. Several patients of this class have fallen to my charge, with whom all injections had to be abandoned, and cold compresses or the ice pack resorted to. These usually afford relief, unless the hemorrhage having continued some time has become aggravated."

Dr. B. then details a case of profuse urethral hemorrhage, occurring in a young colored hostler, after using a mild astringent injection for the relief of a gonorrhœa. The hemorrhage not being controlled by cold compresses, he was ordered fifteen drop doses of fluid ext. ergot every two hours, and rest in bed. After six doses was very much relieved, and a few more doses entirely cured him. The chemico-physiological action of secale cornutum upon the capillaries of the urethra, is analogous to that upon the arterioles of the uterus. D. A. K. S.

Salicylic Acid in Rheumatic Fever.—Dr. Southey.—(*British Med. Journal*, May.) After the first week of the fever, ten grains of the acid dissolved in liquor ammoniæ acetatis is given every two hours for the first twenty-four. It is then given less frequently, so as to produce only slight physiological effects, as noise in the head, etc. By this mode of treatment, the temperature is reduced, arthritis lessened, and the patient rendered less sensitive to the pain. It, however, does not prevent endocarditis, or other complications.

